# Does High C-reactive Protein Concentration Increase Atherosclerosis? The Whitehall II Study

**DOI:** 10.1371/journal.pone.0003013

**Published:** 2008-08-20

**Authors:** Mika Kivimäki, Debbie A. Lawlor, George Davey Smith, Meena Kumari, Ann Donald, Annie Britton, Juan P. Casas, Tina Shah, Eric Brunner, Nicholas J. Timpson, Julian P. J. Halcox, Michelle A. Miller, Steve E. Humphries, John Deanfield, Michael G. Marmot, Aroon D. Hingorani

**Affiliations:** 1 Department of Epidemiology and Public Health, University College London, London, United Kingdom; 2 Medical Research Council Centre of Causal Analyses in Translational Epidemiology, Department of Social Medicine, University of Bristol, Bristol, United Kingdom; 3 Vascular Physiology Unit, Institute of Child Health, University College London, London, United Kingdom; 4 Great Ormond Street Hospital NHS Trust, London, United Kingdom; 5 Department of Epidemiology and Population Health, London School of Hygiene and Tropical Medicine, London, United Kingdom; 6 Department of Medicine, University College London, London, United Kingdom; 7 Clinical Sciences Research Institute, Warwick Medical School, University of Warwick, Coventry, United Kingdom; 8 Centre for Cardiovascular Genetics, University College London, United Kingdom; University of Newcastle, United Kingdom

## Abstract

**Background:**

C-reactive protein (CRP), a marker of systemic inflammation, is associated with risk of coronary events and sub-clinical measures of atherosclerosis. Evidence in support of this link being causal would include an association robust to adjustments for confounders (multivariable standard regression analysis) and the association of *CRP* gene polymorphisms with atherosclerosis (Mendelian randomization analysis).

**Methodology/Principal Findings:**

We genotyped 3 tag single nucleotide polymorphisms (SNPs) [+1444T>C (rs1130864); +2303G>A (rs1205) and +4899T>G (rs 3093077)] in the *CRP* gene and assessed CRP and carotid intima-media thickness (CIMT), a structural marker of atherosclerosis, in 4941 men and women aged 50–74 (mean 61) years (the Whitehall II Study). The 4 major haplotypes from the SNPs were consistently associated with CRP level, but not with other risk factors that might confound the association between CRP and CIMT. CRP, assessed both at mean age 49 and at mean age 61, was associated both with CIMT in age and sex adjusted standard regression analyses and with potential confounding factors. However, the association of CRP with CIMT attenuated to the null with adjustment for confounding factors in both prospective and cross-sectional analyses. When examined using genetic variants as the instrument for serum CRP, there was no inferred association between CRP and CIMT.

**Conclusions/Significance:**

Both multivariable standard regression analysis and Mendelian randomization analysis suggest that the association of CRP with carotid atheroma indexed by CIMT may not be causal.

## Introduction

C-reactive protein (CRP) is a non-specific marker of systemic inflammation, but whether it plays a causal role in atherosclerosis and its complications remains controversial. Findings examining potential direct proatherogenic effects of CRP in vitro and in vivo are mixed [1]–[3]. Randomised controlled trials specific to CRP are currently lacking. Several observational studies show high circulating CRP to be associated with increased risk of coronary heart disease (CHD) events [4]–[6] and increased carotid intima-media thickness (CIMT) [7]–[9], a subclinical marker of atherosclerosis [10], [11]. However, these associations may have non-causal explanations as a result of reverse causality (i.e., CRP levels may be altered as a result of atherosclerosis rather than being a cause of it) or confounding (the association of CRP with atherosclerosis may arise from the common association of the two with other causative factors). Indeed, CRP is related to many other risk factors, such as obesity, smoking and socioeconomic adversity, as well as other “novel” risk factors such as fibrinogen and interleukin-6 [7], [12]–[14].

Recent genetic findings offer opportunities for testing the causal relevance of CRP using the principles of Mendelian randomization [15]–[18]. Common genetic variants have been identified that robustly affect the level of circulating CRP [19]–[21]. Because of their randomized allocation at conception (according to Mendel's Laws), the genetic variants may be used as unconfounded proxies for CRP. The “central dogma” of the unidirectional flow of information from common genome variation → protein → phenotype over the life course also means that reverse causality and effect dilution are overcome in genetic studies. Thus, use of gene variants as an unconfounded instrument for CRP levels offers the opportunity of assessing the causal relevance of CRP for atherosclerosis.

To our knowledge two previous studies have examined the association of genetic variants in the *CRP* gene with CIMT. The Cardiovascular Risk Factors in Young Finns study used variation in the *CRP* haplotypes as an instrument for unconfounded CRP levels and found no association between the CRP instrument and CIMT in young adults aged 24 to 39 years [22]. However, CIMT may not be as appropriate an indicator of atherosclerosis in that age group as in older people [23]. In the US population-based Cardiovascular Health Study, conducted on older adults, there was also no direct associations between *CRP* haplotypes and CIMT although an association of CRP single nucleotide polymorphisms (SNPs) with CHD events was noted in a subgroup [24]. However, the study did not directly evaluate the quantititive associations of *CRP* genotypes, CRP and vascular outcomes for their consistency. An instrumental variables analysis using *CRP* genotypes or haplotypes as a proxy for CRP would inform whether CRP levels are causally associated with CIMT [16].

We sought to investigate the potential for a causal association between CRP and atherosclerosis by standard observational methods of multivariable analyses, adjusting for confounding by other risk factors, and also by using haplotypes from 3 variants in the *CRP* gene as instrumental variables for the unconfounded and unbiased (by reverse causation and regression dilution bias) effect of CRP on CIMT. Analyses were undertaken in a well-established occupational cohort of British civil servants, the Whitehall II study, who were at mean age 49 at the first measurement of CRP and at 61 at the second measurement of CRP. In a companion paper based on Whitehall II and four other cohorts we extend the instrumental variables analysis to disease endpoints by examining the associations between *CRP* genotype, circulating CRP and manifest CHD [25].

## Results

Participants were mostly men and from non-manual position as the latest occupational status (table 1). As expected, *CRP* haplotypes were associated with circulating CRP levels (table 2) explaining in combination 3.9% of the variation in CRP at age 49 years and 3.3% of the variation in CRP at age 61 years. *CRP* haplotypes were not, however, associated with risk factors, such as high blood pressure, dyslipidaemia, obesity, physical inactivity and low socioeconomic position (1 of 36 tests statistically significant at p<0.05; 1–2 would have been expected by chance). In contrast, all of the risk factors were associated with serum CRP and/or CIMT (table 3).

**Table 1:** Participant Characteristics.

Characteristic	Mean (SD)	N (%)	Median (IQR)	Total N
Age, y	61.0 (6.0)			4941
Women		1331 (26.9)		4941
Systolic blood pressure, mm Hg	127 (17)			4939
Diastolic blood pressure, mm Hg	74 (10)			4939
HDL cholesterol, mmol/l	1.58 (0.45)			4939
LDL cholesterol, mmol/l	3.51 (1.75)			4883
Triglycerides, mmol/l	1.39 (0.92)			4939
Body mass index, kg/m^2^	26.7 (4.2)			4920
Physical inactivity		738 (15.1)		4886
Low occupational status*		400 (8.2)		4903
Ever smoking		2369 (48.0)		4938
Prevalent diabetes		329 (7.2)		4583
Prevalent coronary heart disease		410 (8.3)		4941
Serum C-reactive protein, mg/L			1.22 (0.63–2.59)	4941
Previous serum C-reactive protein, mg/L†			0.83 (0.42–1.69)	4435
Carotid intima-media thickness, mm	0.79 (0.15)			3299

* Low occupational status refers to clerical position in a three level hierarchy of administrative, professional and clerical employment grade.

† Measured at mean age of 49.2 (SD = 6.0) years

**Table 2:** Association Between 4 *CRP* Haplotypes and Serum C-reactive Protein (CRP) Concentration.

	Median (IQR) CRP, mg/L	
Haplotype of +1444, +2302 and +4899 SNPs	At mean age 61.0 years (n = 4941)	At mean age 49.2 years (n = 4435)
CAT		
0 (n = 2165 at age 61.0/n = 1955 at mean age 49.2)	1.34 (0.72 to 2.75)	0.91 (0.46 to 1.91)
1 (n = 2249/2008)	1.17 (0.61 to 2.50)	0.78 (0.39 to 1.58)
2 (n = 527/472)	1.00 (0.48 to 2.06)	0.73 (0.37 to 1.36)
*P for trend* *	*<0.0001*	*<0.0001*
CGG		
0 (n = 4423/3971)	1.18 (0.62 to 2.51)	0.80 (0.41 to 1.65)
1 (n = 499/447)	1.50 (0.81 to 2.97)	0.98 (0.56 to 1.93)
2 (n = 19/17)	1.97 (1.13 to 4.67)	1.82 (1.47 to 4.36)
*P for trend* *	*<0.0001*	*<0.0001*
CGT		
0 (n = 2383/2126)	1.25 (0.64 to 2.66)	0.87 (0.44 to 1.80)
1 (n = 2101/1901)	1.19 (0.63 to 2.47)	0.78 (0.40 to 1.62)
2 (n = 457/408)	1.21 (0.69 to 2.69)	0.80 (0.40 to 1.68)
*P for trend* *	*0.74*	*0.006*
TGT		
0 (n = 2333/2091)	1.15 (0.59 to 2.45)	0.78 (0.39 to 1.56)
1 (n = 2189/1968)	1.26 (0.66 to 2.63)	0.84 (0.43 to 1.76)
2 (n = 419/376)	1.50 (0.76 to 2.86)	1.03 (0.55 to 2.45)
*P for trend* *	*<0.0001*	*<0.0001*

* Adjusted for age and sex.

**Table 3:** Contemporaneous Associations of Risk Factors with Serum C-reactive Protein (CRP) Concentration and Carotid Intima-media Thickness (CIMT) at Mean Age 61.0 Years*.

	Log CRP (mg/L)			CIMT (mm)		
Risk factor	N	Beta (95% CI)	*P*	N	Beta (95% CI)	*P*
Systolic blood pressure, mm Hg	4939	0.009 (0.007 to 0.011)	*<0.0001*	3299	0.001 (0.001 to 0.002)	*<0.0001*
Diastolic blood pressure, mm Hg	4939	0.017 (0.015 to 0.021)	*<0.0001*	3299	0.001 (0.000 to 0.001)	*0.002*
HDL-cholesterol, mmol/l	4939	−0.64 (−0.71 to −0.57)	*<0.0001*	3299	−0.027 (−0.039 to −0.015)	*<0.0001*
LDL-cholesterol, mmol/l	4883	0.016 (−0.001 to 0.033)	*0.07*	3266	0.003 (0.001 to 0.006)	*0.008*
Triglycerides, mmol/l	4939	0.21 (0.17 to 0.24)	*<0.0001*	3299	0.005 (−0.001 to 0.011)	*0.10*
Body mass index, kg/m^2^	4920	0.10 (0.096 to 0.11)	*<0.0001*	3291	0.003 (0.002 to 0.005)	*<0.0001*
Smoking†	4938	0.22 (0.16 to 0.28)	*<0.0001*	3297	0.019 (0.009 to 0.029)	*0.0003*
Physical inactivity†	4886	0.18 (0.10 to 0.27)	*<0.0001*	3274	0.000 (−0.014 to 0.015)	*0.96*
Low occupational status†	4903	0.16 (0.04 to 0.27)	*0.009*	3282	0.001 (−0.019 to 0.022)	*0.90*

* Based on age- and sex-adjusted linear regression models.

† Binary variables: 0 = never smoker, 1 = ever smoker; 0 = non-sedentary, 1 = sedentary; 0 = non-manual, 1 = manual.

After adjustment for age and sex, higher contemporaneous and previous serum CRP concentrations were associated with increased CIMT (table 4). However, further adjustment for risk factors attenuated these associations to the null suggesting that risk factors may confound or mediate the association between CRP and CIMT. The total reduction of the magnitude of the CRP-CIMT association between the two models at the mean age of 61.0 was 75%. Of the separate risk factors, adjustment for BMI alone reduced the age- and sex-adjusted association between CRP and CIMT by 57% (p for association = 0.16 after adjustment), the corresponding reduction being 29% for systolic blood pressure, 27% for HDL-cholesterol and less than 15% for other risk factors (p< = 0.01). Excluding those with CRP greater than 10 mg/L (n = 54 at age 49.2; n = 105 at age 61.0) had little effect on the results presented in table 4. The findings were also replicated in a subcohort including only individuals with no CHD or diabetes (n = 2608 for CRP measured at mean age 61, n = 2393 for CRP at mean age 49).

**Table 4:** Associations Between Serum C-reactive Protein (CRP) Measured at Two Time Points and Carotid Intima-media Thickness (CIMT) Obtained from Standard Multivariable Regression Analysis.

		Beta (95% CI) for CIMT (mm) at mean age 61.1	
Exposure	N	Age and sex adjusted	Age, sex and risk factor adjusted*
Per doubling of CRP concentration at mean age of 61.0 years	3225	0.006 (0.003 to 0.009) *P = 0.0004*	0.001 (−0.002 to 0.005) *P = 0.41*
Per doubling of CRP concentration at mean age 49.2 years	2948	0.006 (0.002 to 0.009) *P = 0.0006*	0.001 (−0.002 to 0.005) *P = 0.30*

Only participants with no missing data in any of the covariates are included.

* Adjusted for age, sex, systolic blood pressure, diastolic blood pressure, HDL-cholesterol, LDL-cholesterol, triglycerides, body mass index, smoking, physical inactivity, and low occupational status.

The analysis of haplotypes in the *CRP* gene as instrumental variables for the unconfounded and unbiased effect of CRP on CIMT, was undertaken using two-stage least squares method [16]. All F-statistics from the first-stage regressions in the instrumental variable models were greater than 10 (17.4 for contemporaneous and 16.8 for previous serum CRP) indicating sufficient strength to ensure the validity of instrumental variable methods in these data. The second step of the instrumental variables analysis, consistent with the confounder adjusted standard regression analysis, suggested no association between CRP and CIMT, though this was estimated with wide confidence intervals (table 5). This finding was replicated in a subcohort that included only individuals with no CHD or diabetes (for contemporaneous association at mean age 61 age- and sex-adjusted beta = −0.007, 95% CI −0.039 to 0.25, p = 0.68, N = 2660; the corresponding figures where beta = −0.006, 95% CI −0.039 to 0.027, p = 0.72, N = 2440 for CRP measured at mean age 49 and CIMT at mean age 61).

**Table 5:** Associations of C-reactive Protein (CRP) with Carotid Intima-media Thickness (CIMT) Obtained from the Instrumental Variables Analysis in Which *CRP* Haplotypes Act as An Instrument for the Non-confounded and Unbiased Effect of CRP.

Exposure	N	Age and Sex Adjusted Beta (95% CI) for CIMT (mm) at Mean Age of 61.0 Years
Per doubling of CRP concentration at mean age 61.0	3299	−0.005 (−0.031 to 0.021) *P = 0.71*
Per doubling of CRP concentration at mean age 49.2	3016	−0.001 (−0.025 to 0.023) *P = 0.94*

Finally, levels of CIMT did not vary by *CRP* haplotypes (all p>0.63) suggesting that these haplotypes have no effect on CIMT although they are consistently associated with serum CRP concentrations in middle and late adulthood in this cohort.

## Discussion

Atheromatous plaques start to progress from childhood and may eventually become prone to plaque rupture in adulthood leading to clinical events, such as acute myocardial infarction, unstable angina or stroke. In this large prospective cohort study, both a Mendelian randomization approach, in which confounding is controlled for by using genetic variants as instruments for the unconfounded association, and standard multivariable regression analyses (adjusting for a range of potential confounding factors) were consistent in showing no independent association of CRP with CIMT. These findings could be explained if CRP does not itself contribute to the development of atherosclerosis but rather marks pro-atherogenic exposures, the presence of atheroma, or a combination of the two.

We used haplotypes in the *CRP* gene that were constructed on the basis of tag SNPs rs1205, rs1130864 and rs3093077 that capture comprehensively the common variability at the *CRP* locus in subjects of European descent [19]–[21]. A recent large-scale meta-analysis of genetic association studies of 8 *CRP* polymorphisms and CRP concentration used a novel Bayesian approach that allows integration of informative data from a wide range of studies, irrespective of the specific *CRP* polymorphism typed [26]. All the three SNPs we studied were found to mark haplotypes likely to harbour functional variants in the vicinity of the *CRP* gene that could regulate its level. In the present study, these SNPs were consistently associated with serum CRP levels across two time points 12 years apart suggesting that the haplotypes defined groups with long-term differences in circulating CRP. However, there was no strong statistical evidence that these haplotypes influencing serum CRP levels were related to CIMT after taking into account the magnitude of their association with CRP. This null finding is assumed to represent a non-confounded and unbiased estimate of the association between CRP and CIMT because the existence of early stages of atherosclerosis cannot alter inherited haplotypes [15], and the potential confounders of the CRP-atherosclerosis association (e.g., obesity, smoking, physical inactivity or socioeconomic adversity) were distributed evenly among the different CRP haplotypes.

Our findings are consistent with the null findings in two smaller studies on *CRP* genotypes and CIMT, one related to young adults aged 24 to 39 years [22] and the other to an older cohort than ours [24]. In combination, these and other genetic studies related to less direct correlates of atherosclerosis, such as blood pressure [12] and metabolic syndrome [20] provide evidence against the status of CRP as a causal factor for atherosclerosis. Lange et al. [24] suggest that CRP may affect plaque rupture rather than atherosclerosis in a study reporting an association of *CRP* genotype with incident CHD in a subgroup but no association with CIMT in the same subgroup or in the study population as a whole. However, the association between *CRP* genotype and CHD has not been confirmed by other studies or meta-analyses [19], [25], [27]. A companion for this study is the largest meta-analysis on this issue to date, based on Whitehall II and four other general population cohorts. That study showed no association between a single *CRP* polymorphism and incident or prevalent CHD in a total of 18,637 participants (4,610 cases) [25]. However, a very large sample size (around 20,000 cases and controls), with comprehensive tag SNP typing, such as that being assembled by the CRP-CHD genetics collaboration (CCGC) [28], will be necessary to confirm or refute a causal association of CRP with risk of CHD events.

Several issues may compromise the value of the Mendelian randomisation approach in determining causality [29]. First, such an approach requires the existence of genetic variants that have been shown to be robustly (replicated in several independent studies) associated with the non-genetic modifiable exposure of interest. For the haplotypes that we have used here, such a robust association has been established in multiple independent studies [20], [21], [30]–[38], and was confirmed in our dataset. Furthermore, the association of the haplotype (instrumental variable) was strong enough for the instrumental variables analysis to be consistent as the F-statistic was above the value of 10 suggested as a threshold to distinguish weak vs. strong instruments [39].

Second, population stratification, resulting from factors such as ancestral patterns of geographical migration and differences in mating practices and reproductive behaviors between populations, may confound genotype-phenotype associations and is often speculated to be the reason for non-replication of genetic associations [40]. There is some evidence of such confounding in relation to ethnic groups, i.e., relationships between genotype and phenotype that were found in multiethnic populations disappeared when analysed separately in each ethnic group [41], [42]. Population stratification may not only potentially lead to such false positive genotype-phenotype associations but can also, in principle, mask associations. To increase protection against bias from population stratification we restricted our analyses on white Europeans only. We also confirmed that there was no stratification in *CRP* haplotypes between socioeconomic groups. Furthermore, the null finding of *CRP* haplotype and CIMT is replicable as consistent findings have been obtained from this UK study and studies in a US and Finnish population [22], [24]. For these reasons, it seems unlikely that population stratification would have masked a causal association between CRP and CIMT.

Third, the Mendelian randomisation approach may be compromised if genetic variants used as instruments have multiple effects on phenotype (pleiotropy) or if the variants are in linkage disequilibrium with another genetic variant, that influences the pathway of interest in the opposite direction. We think pleiotropy is unlikely for the variants that we used to generate the *CRP* haplotypes as they are in very close linkage disequilibrium with variation within a putative transcription factor binding site located 5′ of the *CRP* gene that has been associated with circulating concentrations of CRP and thought to be functional [43], [44]. The variants also lie in a block of allelic association that does not contain any other gene with a role in CRP regulation [26], [45].

Fourth, developmental compensation (or canalization) in early life whereby genetically-determined alterations in CRP might be buffered by compensatory changes in other systems may compromise the validity of the Mendelian randomisation approach [46]. However, most recognised examples of developmental compensation relate to dramatic genetic or environmental insults [46] and it is unclear whether the generally smaller phenotypic differences induced by common functional polymorphisms, as used in our study, will be sufficient to induce compensatory responses.

Fifth, the most important limitation is that the instrumental variables analysis provided wide confidence intervals for the effects of CRP on CIMT suggesting that larger samples are needed to obtain more precise estimation. Moreover, CIMT, although a valid non-invasive index of carotid atherosclerosis [11], may not comprehensively capture the general atherosclerotic process.

Nevertheless, despite these limitations, standard multivariable regression analyses of CRP levels and CIMT produced converging support for the conclusions from Mendelian randomization analyses. The association between serum CRP and CIMT attenuated towards the null in adjustments for obesity and other risk factors and this is consistent with several previous studies [7]–[9], [47]. It has been argued that systemic inflammation (of which CRP is a marker) might cause increases in blood pressure, BMI and changes in lipid profiles that might mediate an increase in CIMT and CHD risk [48]. If so, adjustment for these variables in a multivariable model might actually be controlling for factors in the causal pathway. However, adjustment for BMI (which had the most potent attenuating effect) is unlikely to represent an overadjustment, since weight gain is associated with an increase in CRP, and weight loss with a CRP reduction [49]–[51]. Furthermore, *CRP* genotypes that are associated with higher CRP were not associated with BMI, nor with a range of other established or novel risk factors for CHD [19], [20], [22].

In conclusion, the consistency of evidence from both the Mendelian randomisation approach and the multivariable regression analysis approach (each of which has distinct, but differing potential limitations) implies that the association of CRP with CIMT may be better explained by CRP marking the presence of atheroma, or other risk factors rather than having a direct causal role itself, as has been suggested [52]. However, much larger analyses using the genetic approach we and others have described, as well as intervention studies involving a new, specific CRP-inhibitor [53] are needed to more definitively assess the potential causal role for CRP in atherosclerosis and CHD.

## Materials and Methods

### Participants

In 1985, all non-industrial civil servants aged between 35 and 55, in 20 departments in Central London were invited to a cardiovascular medical examination at their workplace [54]. With a 73% participation, the cohort included 6895 men and 3413 women at study entry in 1985–1988. Measurement of CRP was conducted in 1991–1993 and again in 2003–2004 when variants in the *CRP* gene were genotyped and CIMT was assessed. A total of 5949 individuals participated in the latter clinical screening and were successfully genotyped for variants in the *CRP* gene. We excluded non-white subjects (n = 481), those with missing data on haplotypes (n = 13) or CRP concentration (n = 514). Thus, the study sample with complete data on CRP genotype and CRP levels for the cross-sectional analyses in 2003–2004 included 4941 (3610 men and 1331 women) individuals aged 50–74 years (mean age 61.0). We additionally performed prospective analyses with CRP in 1991–1993 as the exposure variable. For these analyses, the study sample comprised 4435 (3255 men and 1180 women) individuals, a sub-group of those included in the cross-sectional analyses, who in addition had measurements of CRP concentration assessed 1991–1993 when they were aged 39–64 years (mean age 49.2 years). Participants included in any analyses provided written informed consent and the study complies with the guidelines of the Declaration of Helsinki.

### Clinical Characteristics

Clinical characteristics included age, sex, systolic and diastolic blood pressure, HDL- and LDL-cholesterol. triglycerides, body mass index (BMI, weight in kilograms divided by height in meters squared), smoking, physical activity, socioeconomic position, and status of diabetes and CHD, all measured in 2003–2004 at mean age 61 years. Systolic and diastolic blood pressure were measured twice using the Hawksley random-zero sphygmomanometer with the participant sitting after a 5-minute rest. The average of these two measures was recorded. Systolic was the pressure at which the Korotkoff sound was first heard clearly and diastolic was the pressure at which the sound disappeared. Blood samples were collected after either an 8-h fast (participants presenting to the clinic in the morning) or at least 4 h after a light fat-free breakfast (participants presenting in the afternoon). Venepuncture of the left antecubital vein was performed with tourniquet. Blood was collected into plain and fluoride Sarstedt (Neumbrecht, Germany) monovettes. Serum for lipid analyses was refrigerated at −4°C and assayed within 72 hours. Cholesterol and triglycerides were measured with the use of a Cobas Fara centrifugal analyzer (Roche Diagnostics System, Nutley, NJ). HDL-cholesterol was measured by precipitating non-HDL cholesterol with dextran sulfate-magnesium chloride with the use of a centrifuge and measuring cholesterol in the supernatant fluid. LDL-cholesterol concentration was calculated using the Friedewald formula. Weight was measured with all items of clothing removed except underwear. A Soehnle scale was used to weigh individuals to the nearest 0.1 kg. Height was measured to the nearest mm using a stadiometer with the participant in bare-feet, standing completely erect with the head in the Frankfurt plane. Smoking (ever smoker vs never smoker) and physical inactivity (sedentary vs not) were recorded. Socioeconomic position was a dichotomy, clerical vs not, based on employment grade in 2003–2004 or, if retired, the latest employment grade.

Diabetes status at mean age 61 was assessed on the basis of 75g oral glucose tolerance test, use of diabetes medication or self-report of doctor diagnosis, all measured at mean ages 49, 56 and 61. Diabetes was defined by 2h glucose≥11.1 mmol/L or fasting glucose≥7 mmol/L. Prevalent CHD comprised a history of non-fatal myocardial infarction or definite angina. Potential prevalent cases of non-fatal myocardial infarction were ascertained by questionnaire items on chest pain [55] and the physician's diagnosis of a heart attack. The confirmation of myocardial infarction according to MONICA criteria [56] was based on electrocardiographic findings, markers of myocardial necrosis and a history of chest pain in the medical records. The assessment of angina was based on the participant's reports of symptoms, with corroboration in medical records or abnormalities in a resting electrocardiogram (ECG), an exercise ECG, or a coronary angiogram.

### CRP Polymorphism Genotyping

DNA was extracted from blood samples obtained at baseline using magnetic beads technology (Geneservice Ltd, Cambridge). Using validated genotype data (minor allele frequency >5%) from subjects of European descent from the NHLBI PGA database (http://pga.mbt.washington.edu/), and the human HapMap database (http://www.hapmap.org/), we examined the pattern of linkage disequilibrium across the *CRP* gene. We then used the haplotype LD r2 method to select a set of tagging (t)SNPs capable of capturing maximum haplotype diversity among subjects of European descent using the programme TagIT (http://popgen.biol.ucl.ac.uk/software.html). We genotyped 3 SNPs in the *CRP* gene [+1444T>C (rs1130864); +2303G>A (rs1205) and +4899T>G (rs 3093077)] using the ABI Prism 7900HT Sequence Detection System for both PCR and allelic discrimination (Applied Biosystems, Foster City, CA). The SNPs were genotyped using Assays by Design from Applied Biosystems under standard conditions. Genotype calling was done manually from the PCR run component tab. The Hardy Weinberg Equilibrium (HWE) was tested at each SNP and CRP +2303 and +4899 were found to be in HWE (χ^2^ p>0.05), but +1444 was not in HWE (p = 0.003). The +1444 SNP was re-genotyped from 678 samples in a different laboratory and the results called by a researcher who was blind to the original results. The mismatch rate was 0.5% suggesting that lacking HWE for +1444 may be due to random residual genotyping error, but biological selection bias or other populational inhomogeneity cannot fully be excluded.

### Measurement of C-Reactive Protein

CRP was measured in serum stored at −80°C using a high-sensitivity immunonephelometric assay in a BN ProSpec nephelometer (Dade Behring, Milton Keynes, UK). Values below the detection limit (0.154 mg/L) were assigned a value of 0.077 mg/L (n = 333 (7.1%) in 1991–1993 at mean age 49 and n = 104 (2.0%) in 2003–2004 at mean age 61). Samples from both study phases were analyzed at the same time. Intra- and inter-assay coefficients of variation were 4.7% and 8.3%. To measure short-term biological variation and laboratory error, a repeated sample was taken from a subset of 150 participants in 1991–1993 and 533 participants in 2003–2004 (average time between samples 32 (SD = 10.5) and 24 (SD = 11.0) days respectively). Reliability between samples was assessed with intraclass correlation: r = 0.83 in 1991–1993 and r = 0.57 in 2003–2004.

### Measurement of Carotid Intima-media Thickness

Ultrasound vascular measurements in 2003–2004 were taken in a temperature controlled (22–26 degrees centigrade), quiet room using a non-invasive, high- resolution ultrasound system, the Aloka Prosound 5500 with a 7.5 MHz linear array transducer. Participants were examined in a supine position, with the head turned to a 45 degree angle away from the side to be scanned. CIMT was measured in the right and left common carotid arteries. Longitudinal images of the common carotid artery, triggered on the R-wave of the ECG, were magnified and recorded in DICOM format as a cine loop, on the hard drive of the ultrasound machine for later analysis. The common CIMT was measured at its thickest part 1 cm proximal to the bifurcation. A measurement was taken between the leading edge of the intima and the media adventitia on 3 separate images on each side using electronic callipers and the mean of the 6 measures was used for analysis. Three observers conducted CIMT studies with inter and intra-observer variability measurements ranging between 2.6% and 5.8%. The overall coefficient of variation for repeated measures of CIMT was 4.7% (N = 89).

### Data Analysis

#### Standard Regression Analysis

We used age- and sex-adjusted least square regression analysis to assess (i) the associations between potential confounding factors (BMI, smoking, physical activity and socioeconomic position) and circulating CRP levels and between potential confounding factors and CIMT; (ii) the association between circulating CRP levels and CIMT (in a multivariable model, additional adjustment was made for potential confounding factors); and (iii) the association of haplotypes (see below) with circulating CRP levels, potential confounding factors and CIMT. The haplotype-confounder associations were undertaken to test our underlying hypothesis that genetic variants in CRP would not be associated with other risk factors that affect conventional observational epidemiological associations.

#### Haplotype Construction

We constructed haplotypes with the genetic data analysis program SIMHAP (see http://www.genepi.com/au/project/simhap, obtained May 2, 2007), using 1000 iterations and a posterior probability >0.95. With this procedure, only one haplotype pair was constructed for each participants. The 13 individuals with a haplotype with a frequency less than 1% were not included in our cohort of 4941 individuals. Thus, 4 haplotypes of SNPs +1444, +2302 and +4899 (CAT, CGG, CGT and TGT) remained in the analysis in which genetic variants were used to determine the association of CRP with CIMT.

#### Instrumental Variables Analysis

An instrumental variables analysis, in which haplotypes in *CRP* were used as instrumental variables for the unconfounded and unbiased effect of CRP on CIMT, was undertaken using two-stage least squares method [16]. In these analyses we used a model for the haplotype-CRP association that assumes each of a participant's two haplotypes contributes additively to his/her value of CRP, as done in a previous study that used similar *CRP* haplotypes as instruments for the effect of CRP on components of the metabolic syndrome [20]. We used the F-statistics from the first-stage regressions to evaluate the strength of the instruments (values greater than 10 are taken to indicate sufficient strength to ensure the validity of instrumental variable methods) [39]. Instrumental variable regression analysis was performed with Stata, version 9.2 (Stata Institute, Texas, USA).

#### General Analytic Procedures

There was no strong statistical evidence that any of the associations we examined differed by sex, which is consistent with previous studies in the field [22], [24]. Therefore all results are presented for women and men combined. Due to skewness, we logarithmically transformed CRP in the analyses, we used logs to base 2 so that we could present associations per doubling of CRP, which are easy to interpret and consistent with previous studies in this area [20], [22]. All analyses (except haplotype construction and instrumental variable analysis) were performed with SAS statistical software, version 9.1 (SAS Institute, Cary, USA).
